# An International Consensus Definition of the Wish to Hasten Death and Its Related Factors

**DOI:** 10.1371/journal.pone.0146184

**Published:** 2016-01-04

**Authors:** Albert Balaguer, Cristina Monforte-Royo, Josep Porta-Sales, Alberto Alonso-Babarro, Rogelio Altisent, Amor Aradilla-Herrero, Mercedes Bellido-Pérez, William Breitbart, Carlos Centeno, Miguel Angel Cuervo, Luc Deliens, Gerrit Frerich, Chris Gastmans, Stephanie Lichtenfeld, Joaquín T Limonero, Markus A Maier, Lars Johan Materstvedt, María Nabal, Gary Rodin, Barry Rosenfeld, Tracy Schroepfer, Joaquín Tomás-Sábado, Jordi Trelis, Christian Villavicencio-Chávez, Raymond Voltz

**Affiliations:** 1 School of Medicine and Health Sciences, Universitat Internacional de Catalunya, Barcelona, Spain; 2 Nursing Department, School of Medicine and Health Sciences, Universitat Internacional de Catalunya, Barcelona, Spain; 3 Palliative Care Service, Institut Català d’Oncologia, Bellvitge Biomedical Research Institute (IDIBELL), Barcelona, Spain; 4 Unidad de Cuidados Paliativos, Hospital Universitario La Paz, Madrid, Spain; 5 Institute of Health Research Aragon, Cátedra de Profesionalismo y Ética Clínica, Universidad de Zaragoza, Zaragoza, Spain; 6 Escola Universitària d’Infermeria Gimbernat, Autonomous University of Barcelona, Barcelona, Spain; 7 Memorial Sloan-Kettering Cancer Center, New York, NY, United States of America; 8 ATLANTES Research Program, Institute for Culture and Society and Palliative Medicine Department, Clinica Universidad de Navarra, University of Navarra, Navarra, Spain; 9 Complejo Hospitalario Infanta Cristina, Badajoz, Spain; 10 End-of-Life Care Research Group, Ghent University & Vrije Universiteit Brussel, Brussels, Belgium; 11 Department of Palliative Medicine, University Hospital of Cologne, Cologne, Germany; 12 Catholic University of Leuven, Leuven, Belgium; 13 Ludwig-Maximilians-Universität München, Munich, Germany; 14 Faculty of Psychology, Stress and Research Group, Universitat Autònoma de Barcelona, Bellaterra, Barcelona, Spain; 15 Department of Philosophy and Religious Studies, Faculty of Humanities, Norwegian University of Science and Technology (NTNU), Trondheim, Norway; 16 Palliative Care Supportive Team, Hospital Universitario Arnau de Vilanova, Lleida, Institut Català de la Salut, IRB, Lleida, Spain; 17 Department of Supportive Care, Princess Margaret Cancer Centre, Department of Psychiatry and Global Institute Psychosocial, Palliative and End-Life Care (GIPPEC), University of Toronto, Ontario, Canada; 18 Department of Psychology, Fordham University, Bronx, New York, United States of America; 19 School of Social Work, University of Wisconsin-Madison, Wisconsin, United States of America; Cardiff University, UNITED KINGDOM

## Abstract

**Background:**

The desire for hastened death or wish to hasten death (WTHD) that is experienced by some patients with advanced illness is a complex phenomenon for which no widely accepted definition exists. This lack of a common conceptualization hinders understanding and cooperation between clinicians and researchers. The aim of this study was to develop an internationally agreed definition of the WTHD.

**Methods:**

Following an exhaustive literature review, a modified nominal group process and an international, modified Delphi process were carried out. The nominal group served to produce a preliminary definition that was then subjected to a Delphi process in which 24 experts from 19 institutions from Europe, Canada and the USA participated. Delphi responses and comments were analysed using a pre-established strategy.

**Findings:**

All 24 experts completed the three rounds of the Delphi process, and all the proposed statements achieved at least 79% agreement. Key concepts in the final definition include the WTHD as a reaction to suffering, the fact that such a wish is not always expressed spontaneously, and the need to distinguish the WTHD from the acceptance of impending death or from a wish to die naturally, although preferably soon. The proposed definition also makes reference to possible factors related to the WTHD.

**Conclusions:**

This international consensus definition of the WTHD should make it easier for clinicians and researchers to share their knowledge. This would foster an improved understanding of the phenomenon and help in developing strategies for early therapeutic intervention.

## Introduction

Over the last two decades the phenomenon of the desire for hastened death has attracted growing interest in the health literature. However, conceptualizing this desire or wish to hasten death (WTHD) and establishing its scope is difficult due to a lack of clarity regarding both clinical and terminological aspects [1–4]. In fact, there has yet to be a formal attempt to define the concept, and most clinical studies on this issue do not clearly describe the target phenomenon or the conceptual framework being applied [1, 2]. Overall, the lack of precision and consistency in the terminology used makes it difficult to interpret and draw conclusions from research in this field [5].

In the palliative care (PC) literature, terms such as ‘wish’ or ‘desire’ ‘to die’ or to ‘hasten death’ or other related expressions are used interchangeably [6–11], which is understandable given that their meanings in everyday speech are largely indistinguishable [3]. However, reference is sometimes made to someone ‘considering’, ‘intending’ or ‘deciding’ to die or hasten death without any consideration of the possible nuances involved, that is, of the difference between thoughts of dying in the context of suffering and a genuine wish to die or to hasten death [3]. To complicate matters further, researchers sometimes refer to a ‘request’ to die or to hasten death, or resort to terms that have a more specific meaning in this context, such as assisted suicide or euthanasia^3^, despite there being important differences in what these situations imply at the clinical, ethical, legal and social levels. It should also be noted that the literature often fails to make the necessary distinction between the kinds of situations referred to by the aforementioned terms and a scenario in which a person simply expresses an acceptance of death or, as a result of his or her spiritual or religious beliefs, calmly contemplates a better life ahead [2, 5].

In addition to these terminological issues, the subjective nature of the WTHD and the circumstances surrounding it may not only have different meanings for different patients but also meanings that change over time for a given individual [12]. Indeed, any one patient may experience numerous, partial or even, at times, contradictory wishes [13–15]. Although important conceptual advances have been made in recent years [2, 12, 16–19], the complexity of the issue and the ambiguity affecting the terminology used have posed a problem not only for clinical studies of the prevalence or aetiology of the phenomenon, but also for research into its ethical, legal and social aspects. In an attempt to improve understanding of the WTHD, an international workshop involving experts from 15 European institutions was held in Barcelona in November 2013. In line with a point made by a number of authors [2, 3, 20], this workshop ratified the need for greater conceptual precision regarding the WTHD, and work began on developing an operational definition that would allow better communication both within and between multidisciplinary groups of researchers and clinicians.

The ultimate aim of this work is to facilitate research in this area and to use the knowledge gained to improve the care offered to patients in such circumstances. In this context, the present paper describes the process towards an internationally agreed operational definition of the WTHD, one that could help to overcome some of the limitations described above.

## Material and Methods

The process involved three phases: 1) review of the literature and discussion within the Steering Group (SG), comprising three researchers [AB, CMR and JPS], who coordinated the whole process, 2) a modified nominal group process with European experts, and 3) a modified Delphi process involving participants from around the world. The modified Delphi process was similar to the full Delphi in terms of procedure and intent, the main difference being that we started from a set of selected statements that had been drafted on the basis of a literature review and the results obtained in the nominal group process. This study was approved by the ethics committee of Universitat Internacional de Catalunya.

### Literature Review

The SG began by carrying out an exhaustive review of the literature in order to establish key aspects related to the study phenomenon [1–4]. The search strategy used in PubMed included the following keywords: “wish to die”, “wish to hasten death”, “desire to die”, “desire for death” and “desire for early death”.

The extensive literature review covered a range of research perspectives on the WTHD, including primary qualitative studies that analysed the phenomenon from the patient’s point of view, [14, 16, 19, 21–25], mixed methods research [7], studies that explored the views of health professionals [26–28] and the analysis of systematic reviews of the topic [3, 4, 19]. This enabled us to establish an initial conceptual framework that served as a starting point for the following two stages of the research. The literature consistently indicated that in the context of advanced physical disease, the desire to die has a multifactorial origin and that it is principally determined by psychological, social, spiritual and existential factors [9, 10, 29–34].

### Modified Nominal Group Process

International participants with research and clinical profiles were selected from various European countries and disciplines to ensure heterogeneity. The nominal group (NG) was then conducted in Barcelona in November 2013 according to a predetermined schedule involving four stages [35]. (See Table 1).

**Table 1:** Nominal group methodology.

Predetermined schedule of the Nominal Group conducted.		
Nominal Group Stages		Explanation of each stage
**Stage 1**	Generation of ideas	The objectives of the session were set out
		A summary was offered of current knowledge about the WTHD
		Questions were posed to generate ideas and debate
**Stage 2**	Discussion	Aims:
		Clarify ideas generated in stage 1
		Explore opinions
		Add further proposals
		Care was taken to ensure that each participant felt that his or her contributions were valued
		For the purposes of future study and discussion by the SG, the content of this session was written up
**Stage 3**	Summary and conclusions	Participants were asked to consider any additional ideas that arose after hearing the views of others
		In order to generate new ideas, all the participants’ contributions were discussed
**Stage 4**	Individual prioritization	All the participants were asked to prioritize in writing the main conclusions resulting from the process so far
		Those participants who wished to present their prioritized conclusions to the group as a whole were given the opportunity to do so

### Modified Delphi Process

This technique is based on sequential questionnaires that are completed individually and anonymously by a panel of experts with the aim of reaching a consensus. The key strengths of this approach are the anonymity of participants, structuring of the information flow and provision of regular feedback coordinated by the SG [36, 37]. Using the preliminary results obtained in the literature review and NG processes, we employed a Delphi process involving three rounds.

Participants in the Delphi process were selected by means of intentional sampling. Specifically, we drew up a list of potential participants with reputable experience in the area of study and fulfilled at least one of the following criteria: health professionals with clinical experience in the field of PC, or researchers with knowledge and/or experience related to the WTHD. In order to achieve a high-quality Delphi process, a heterogeneous group of experts was recruited specifically, we selected participants from across various geographical regions (Australia, Belgium, Canada, Germany, The Netherlands, Norway, Spain and the USA,) and from different professional fields (Table 2). Potential participants were contacted by email and informed about the aims of the study, the tasks involved and the estimated necessary time commitment they would need to make.

**Table 2:** Key characteristics of experts participating in all three rounds of the Delphi process.

Round	Gender	Professional background (all of them researchers in palliative care)	Countries represented	Total Number of institutions involved
1, 2 and 3	5 female	Ethicist: 2	Belgium	19
	19 male	Nurse: 2	Canada	
		Philosopher: 1	Germany	
		Psychologist: 5	Netherlands	
		Psychiatrist: 2	Norway	
		Palliative care physician: 9	Spain	
		Social worker: 1	USA	
		Sociologists: 2		

### Questionnaire Development and Delphi Process

Based on the literature review, the workshop contributions and the views of the SG, a preliminary set of key concepts to be included in a definition of the WTHD was drawn up. In order to facilitate discussion of these ideas, a questionnaire was developed that would enable participants to give their opinion about each of the concepts included in the proposed definition and, in particular, the wording of the definition as a whole. To this end, the questionnaire established a preliminary definition of the WTHD, as well as a breakdown of each of the statements or concepts included in it. The task for each participant was to rate their agreement with each proposed statement on a five-point scale (from 5 = strongly agree to 1 = strongly disagree). Space was also provided for participants to make comments and/or suggest an alternative wording for each proposed statement or concept. Likewise, they could suggest an alternative complete definition and/or make general comments.

**Fig 1:**
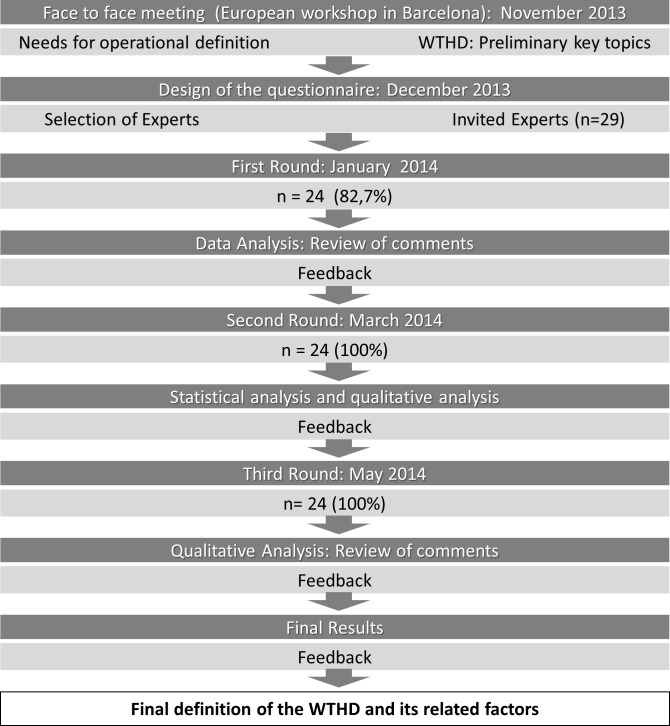
Flowchart of the Delphi process.

This preliminary questionnaire was piloted by sending it to two experts in the field. The questionnaire was sent by email to the panellists chosen for the first round of the Delphi process. The SG had previously established that this process would include two or three rounds, and that for a statement to be accepted, a minimum of 70% agreement would need to be reached among the panellists. The results from this first round were collected by the SG and a revised version of the definition was drawn up based on the responses received. Using the same questionnaire, this revised version was then sent to the panellists for further rating. To facilitate this task, the results from the first round (comments, individual ratings, and percentage agreement reached) were anonymized and shared with participants via a secure cloud-based system. This same procedure of feeding back the results prior to further rating was followed between rounds two and three. Fig 1 shows the process followed in reaching a consensus.

### Statistical Analysis

Data were analysed by two members of the research team (AB, CMR). Questionnaire responses were entered into Microsoft Excel spreadsheets, and the weighted targeted agreement for each statement was calculated using the algorithm proposed by Tastle and Wierman [38].

## Results

### Nominal Group (NG)

Seventeen professionals from 15 European institutions in Belgium, Germany, The Netherlands, Norway and Spain participated. The group debate enabled those involved to share knowledge, to develop a greater understanding of the phenomenon and to reach a number of key agreements. During the NG process, participants acknowledged that there is a lack of conceptual precision regarding the wish to die [1–3, 5], and they agreed that this underlined the need to develop an operational definition that would allow better communication both within and between groups of researchers and clinicians. A number of other shared conclusions were reached by participants during the debate (Table 3).

**Table 3:** Conclusions reached during the Nominal Group.

1.	A useful definition would be acceptable to a heterogeneous group of professionals, from different disciplines and countries
2.	The definition should be reserved for patients with a predominantly physical illness or condition
3.	The wish to die being referred to should be linked to suffering. Said suffering could have several different dimensions
4.	The definition might be applicable to a wide range of patients but its scope should be clearly set out so as highlight those situations in which it would not apply, for example, an ‘*acceptance of death*’.

The group also agreed that further work of the kind they had been engaged in would be necessary, and that the inclusion of new participants from different cultures and professional backgrounds would extend the scope of the study. In this context it was decided that an internet-based Delphi process would be an ideal way of reaching a definition that fulfilled each of these characteristics.

### Modified Delphi Process

Of the 29 experts who were initially contacted, 24 agreed to participate; of these, 15 had previously taken part in the NG. All 24 experts completed the three rounds of the Delphi process. Table 2 shows the key participant characteristics of experts in all three rounds of the Delphi process.

Table 4 shows the percentage agreement reached for each concept/statement after each round, as well as the proposed changes based on the panellists’ comments.

**Table 4:** The 12 statements included in the proposed definition of the WTHD, the percentage agreement for each, and the changes to their wording across the three rounds of the Delphi process.

Statements First Round Delphi Questionnaire		Agreement on Round 1 Wording	Statements Second Round Delphi Questionnaire		Agreement on Round 2 Wording	Statements Third Round Delphi Questionnaire	
Title: Definition of the WTHD			Title: Definition of the WTHD plus addendum			Title: Definition of the WTHD and its related factors	
1	WTHD is a reaction to global suffering	76.4%	1	The WTHD is a reaction to suffering,	92.3%	1	The WTHD is a reaction to suffering,
2	in the context of a severe illness	74.3%	2	in the context of a life-threatening condition,	82.5%	2	in the context of a life-threatening condition,
3	from which the patient can see no way out other than to accelerate his or her death.	84.2%	3	from which the patient can see no way out other than to accelerate his or her death.	79.7%	3	from which the patient can see no way out other than to accelerate his or her death.
4	This complex feeling,	78.1%	4	This wish	96.6%	4	This wish
5	may be expressed spontaneously or after being asked about it,	91.4%	5	may be expressed spontaneously or after being asked about it,	95.7%	5	may be expressed spontaneously or after being asked about it,
6	but it must be distinguished from the peaceful acceptance of impending death	87.4%	6	but it must be distinguished from the acceptance of impending death	92.9%	6	but it must be distinguished from the acceptance of impending death
7	or from a vague wish to die naturally, although preferably soon	77.6%	7	or from a wish to die naturally, although preferably soon.	93.3%	7	or from a wish to die naturally, although preferably soon.
8	The WTHD is related to a combination of several factors	77.6%	8	ADDENDUM: The WTHD may arise in response to one or several factors,	90.2%	8	The WTHD may arise in response to one or more factors,
9	including unrelieved/exacerbation of physical symptoms (e.g., pain, dyspnoea),	77.5%	9	including physical symptoms (either present or foreseen),	85,7%	9	including physical symptoms (either present or foreseen),
10	unrelieved mental/psychological disorder (e.g., depression, hopelessness)	81.4%	10	psychological distress (e.g. depression, hopelessness, fears, etc.),	93.0%	10	psychological distress (e.g. depression, hopelessness, fears, etc.),
11	existential distress (e.g., loss of meaning in life),	86.6%	11	existential suffering (e.g. loss of meaning in life),	94.0%	11	existential suffering (e.g. loss of meaning in life),
12	and fears.	75.8%	12	and social aspects (e.g. feeling that one is a burden).	93.2%	12	or social aspects (e.g. feeling that one is a burden).

#### First round

At the end of this round, the overall agreement reached was 80.7%. None of the individual concepts/statements yielded agreement below the 70% cut-off established by the SG; therefore, it was decided that all 12 statements would be included in the following rounds. The specific analysis of each concept, however, led to a number of changes to the wording of statements 1, 2, 4, 6 and 7 (Table 4). The decision to make these changes was based on the percentage agreement reached, and on the experts’ comments regarding more qualitative aspects.

In this round, four of the panellists argued that the definition did not need to mention the factors related to the emergence of the WTHD (concepts 8 to 12). Upon reflection, however, the SG decided to maintain the reference to these factors but in a separate paragraph headed ‘*Addendum*’ (see Discussion). In addition, the views of these panellists were made clear in the feedback in order to gather the opinions of all participants in the next round.

#### Second round

By the end of this round, the overall agreement had reached 90.8%. Agreement was above 80% for all the individual concepts, with the exception of statement 3 (“from which the patient can see no way out other than to accelerate his or her death”); here, two panellists expressed doubts about whether the word ‘see’ or ‘feel’ should be used, alluding to the extent to which this aspect of the phenomenon was rational or emotional), which reached 79.7%, still well above the minimum established.

The qualitative analysis of Round 1 led to the SG making two decisions in relation to the following. First, the analysis of the panellists’ comments showed that most were in favour of maintaining the reference to possible causal or related factors. Consequently, the SG decided against putting this information in a separate paragraph headed ‘*Addendum*’. The solution chosen instead was to change the title of the proposal to ‘Definition and related factors’ and to include all the information in a single main text, thereby avoiding a partial use of the definition that would undermine its value. Second, statement 2, “in the context of a life-threatening condition”, generated debate among the panellists, some of whom felt it was too restrictive and would not be applicable to situations such as a sudden impairment of functioning or to people who were simply tired of living. Since “life-threatening condition” is the precise term that is generally used to define patients receiving PC, the consensus of the SG was towards maintaining this expression.

#### Third round

All the panellists expressed agreement with the final definition, as well as with the proposed title (Definition of the WTHD and its related factors). Most of the comments in this round, which produced several nuances of opinion, focused on statement 2 (“in the context of a life-threatening condition”), but it was agreed that its inclusion helped link the definition more closely to the PC context in its wider sense. A number of other isolated comments were made by some panellists and these are addressed in the Discussion below.

### Final Definition of the WTHD and its Related Factors

The WTHD is a reaction to suffering, in the context of a life-threatening condition, from which the patient can see no way out other than to accelerate his or her death. This wish may be expressed spontaneously or after being asked about it, but it must be distinguished from the acceptance of impending death or from a wish to die naturally, although preferably soon.

The WTHD may arise in response to one or more factors, including physical symptoms (either present or foreseen), psychological distress (e.g. depression, hopelessness, fears, etc.), existential suffering (e.g. loss of meaning in life), or social aspects (e.g. feeling that one is a burden).

## Discussion

The process of reaching a consensus regarding an operational definition of the wish to hasten death (or ‘desire for hastened death’, since the terms ‘wish’ and ‘desire’ have been used synonymously throughout the study) has been both laborious and revealing. Each word of the definition has been considered at some point or other during the discussions, and this suggests that professionals in this field regard the creation of a consensus definition as being of importance. It should be noted that the proposed definition focuses on the ‘desire’ or ‘wish’ to hasten death, and not on euthanasia, physician-assisted suicide or actions such as the voluntary refusal of food and fluids. Indeed, our aim was precisely to explore in greater depth the conceptualization of this broader issue, that is, the WTHD, which may precede these other more specific acts. In our view, it is this issue which is of greater interest from a clinical point of view. Thus, although further efforts are required, we believe that the work described in this paper will help to refine existing concepts and facilitate greater understanding among and between clinicians and researchers, thus opening up new and important avenues for future investigation.

### Aspects of the consensus definition that should be highlighted

The first point to note is that the WTHD is defined as a reaction to suffering. Although there was some debate over whether the extent of suffering should be specified, for example, by including an adjective such as ‘extreme’, ‘overwhelming’ or ‘unbearable’, the final decision was that this was not required. It was argued that not all cases would necessarily involve suffering of this kind. Furthermore, confusion could arise from the use of the word ‘unbearable’, due to its association with legal initiatives related to assisted suicide or euthanasia. The inclusion of other adjectives such as ‘global’ or ‘multidimensional’ was also debated, but once again it was decided that this was unnecessary, since intense suffering inevitably affects all dimensions of the human individual [39].

A second point is that the definition is circumscribed to people with significant physical illness or incapacity. Although the purpose of the definition was not to specify differences between the WTHD and suicidal ideation, which in fact would be difficult to distinguish, it was agreed that the term WTHD should specifically be used when such a wish is experienced by sick people with limited life expectancy, or by patients requiring PC in the widest sense. In this regard, there was also some deliberation over whether the definition should include the expression ‘life-threatening condition’ or ‘life-limiting condition’. Initially, the term ‘limiting’ (with its implicit reference to both time and physical limitations) was felt to be adequate, but it was eventually decided that ‘life-threatening condition’ was closer to the terminology used in existing definitions of PC, such as that established by the World Health Organization [40]. Although the consensus view among panellists was that the definition should apply strictly to the context of significant physical illness or disability, this does not rule out the possibility that future revisions might see its scope being extended to cover older people who report being ‘tired of living’ in the absence of a life-threatening condition. As is clear, however, from a recent systematic review [41], only a small number of empirical studies have examined this issue in older people, and even fewer have analysed the phenomenon in the absence of mental disorder or a life-threatening condition.

Another feature that emerged during the process of producing the definition was that the WTHD might be present even if not explicitly expressed by the patient. The suggestion to include this aspect was accepted in all three rounds of the Delphi process, and it highlights the perceived clinical importance of detecting the phenomenon and of asking the patient about it, even if he or she has not openly expressed such a wish.

The final part of the first paragraph of the proposed definition draws attention to two situations that would not be regarded as synonymous with a WTHD. The first situation would be an acceptance of death, in the hope that it would be peaceful and without suffering, or with a degree of suffering that would be bearable or accepted. This situation would include those patients who see death as a natural process in the context of their condition, that is, something accepted but not actually desired. The second situation is a wish to die without an accompanying wish to hasten death. Wishes of this kind may be associated with the hope of a better life after this one (spiritual, religious or philosophical beliefs, etc.), or with ideas of relief or rest, but without any serious plans to hasten death.

A further point to note is that differentiating between the WTHD and suicidal ideation in people with evident psychiatric, psychological or emotional problems does not seem possible, especially given that severe physical illness is known to be a risk factor for suicide attempts among those with suicidal ideation [17, 42–45]. In this respect, the WTHD would be an overarching term that would include suicidal ideation as one type of such a wish or, in the case of suicide, as an action related to it. Given the complexity of this issue, it is unsurprising that the distinction between suicidal ideation and the WTHD was a question that generated considerable debate among panellists. It is worth noting, however, that it was the experts from the field of psychiatry who were most against the idea of a clear distinction being drawn between the two concepts. What is clear is that it is important to rule out depression or another mental disorder when seeking to identify the presence of a WTHD [2, 44].

### Multifactorial nature of the WTHD

The second paragraph of the proposed definition emphasizes the multifactorial nature of the phenomenon and draws attention to some of the aspects most commonly observed in conjunction with the WTHD. The opportunity to include a mention of these ‘related factors’ was another issue that provoked an interesting debate during the Delphi process. At one point it was argued that, strictly speaking, this fragment need not form part of the definition. However, the final decision was to include a reference to related factors as this helps to specify the concept of the WTHD and to remind clinicians about aspects that should be explored in terms of their possible contribution to such a wish. The aim in mentioning these factors was not to be exhaustive but simply to draw attention to potentially key aspects underpinning the WTHD. While some of the factors included in the definition underwent minor changes as a result of comments made during the three Delphi rounds, the panellists invariably agreed upon the need to mention ‘existential suffering (e.g. loss of meaning in life)’, even though the literature contains no precise definition of what this means in practice [46, 47].

During the Delphi process there was also discussion over whether this final section should include mention of possible meanings that the wish to die might have for the patient, for example, fear (of symptoms getting worse or of the process of dying), a cry for help or the desire to gain some control over life [4, 12, 15, 34]. However, it was ultimately decided that the definition would be more concise.

The possibility of including the content of the second paragraph as an Addendum rather than as a main part of the definition was also considered. The conclusion, however, was that it would be better to retain this content as part of the overall statement and change the title of the latter accordingly, that is, to ‘Definition of the Wish to Hasten Death and its Related Factors’. By including both paragraphs within a single text, there was no risk that the definition might be undermined as a result of only a part of it being taken into consideration. This decision was viewed particularly positively by panellists with a more clinical professional role.

Mention should also be made of certain aspects or finer details that, despite the overall consensus reached, proved to be somewhat controversial and saw different views being expressed. For example, in relation to the concept/statement “from which the patient can see no way out other than to accelerate his or her death”, some panellists were reluctant to use the expression “can see”, which they felt could be interpreted as the outcome of a rational process (i.e. after the analysis of all options) rather than as a more emotional impulse, one linked to feelings. Although the possibility of using the expression “can feel” was considered, it was finally decided that the most practical solution was to retain the original wording (“can see”), since the general view was that, in reality, both feeling and reason can be implicated in such a wish.

Different opinions were also expressed regarding whether the definition should reference the potentially fluctuating or ambivalent nature of the WTHD, although ultimately it was decided not to mention these nuances in the definition.

In our view, the present study has a number of strengths. The process followed is an appropriate way of reaching agreement over a definition of the WTHD that could be widely accepted. Indeed, the methodology used (NG and Delphi process, preceded by an exhaustive literature review) has been applied in similar studies conducted in various disciplines [48–52], and has contributed to the development of common frameworks and working standards that are crucial for advances in biomedical science. The process here involved experts from different countries and various professional disciplines who had a range of experience in relation to the topic in question. Some had a more clinical background, while others were more research-based. This variety offers the possibility of combining different views regarding the phenomenon.

We also acknowledge that the study had limitations. The study is perhaps limited by the relatively small number of participants (n = 24) in the Delphi process, many of whom had already taken part in the NG. A further limitation relates to the lack of participating experts from Asia, Africa or the Middle East, given that there may be cultural differences in attitudes towards hastened death. Although the debate would clearly have been enriched by the participation of a larger number of experts, who may have brought other opinions or discrepant views that are not represented here, we nonetheless believe that the most significant opinions are likely to have been covered, making the exercise both participatory and operative.

### Implications for practice and for research

It should be emphasized that the proposed definition is primarily aimed at—and developed from within—the PC context. PC is understood here in the broad sense, as a multidimensional health intervention that many patients, especially those with chronic illness or degenerative disease, may require at some point in their lives [40]. In this context, a better understanding of the WTHD and a common language for describing it may improve communication both among professionals and between patients and professionals when seeking to address the phenomenon. Distinguishing and defining the WTHD more clearly will also help in terms of its early detection, and should highlight for all those involved in the care of these patients the importance of exploring such a wish and identifying both the meaning it has for a given individual and the factors related with it. However, this will also bring a new set of challenges, since professionals will need to develop the communication skills required to explore and tackle this issue, and individualized treatment protocols will need to be drawn up to reduce the suffering of patients. In this respect, the suffering linked to the social, psychological and existential dimensions (especially the latter) is perhaps the most complex challenge that now needs to be addressed in this context [53].

With respect to research in this field, a unified definition should facilitate a better conceptualization of the phenomenon and lead to an improved understanding of it. Greater clarity about the construct is likely to enable better communication both within and between groups of researchers. This improved communication should lead to descriptions of study populations and definitions used in research that are more precise and, therefore, make it easier to compare and even combine results. The close examination of the WTHD carried out in the present study highlights the need for further research in order to enable its early detection, to identify and assess factors associated with it, and to evaluate the response to therapeutic interventions. Given that conceptual clarity is crucial for ethical and legal research, these fields should also benefit from the work reported here. Finding ways of easing end-of-life suffering expressed as a WTHD is a scientific, professional and human duty, and the consensus definition reached should go some way to achieving this goal.

## References

[1] Rosenfeld B. Assisted suicide, depression, and the right to die. Psychol Public Policy Law. 2000; 6:467–488. 12661536 10.1037/1076-8971.6.2.467

[2] Breitbart W, Rosenfeld B, Pessin H, Kaim M, Funesti-Esch J, Galietta M, et al Depression, hopelessness, and desire for hastened death in terminally ill patients with cancer. JAMA. 2000; 284:2907–2911. 11147988 10.1001/jama.284.22.2907

[3] Monforte-Royo C, Villavicencio-Chávez C, Tomás-Sábado J, Balaguer A. The wish to hasten death: A review of clinical studies. Psychooncology. 2011; 20:795–804. 10.1002/pon.1839 20821377

[4] Monforte-Royo C, Villavicencio-Chávez C, Tomás-Sábado J, Mahtani-Chugani V, Balaguer A. What lies behind the wish to hasten death? A Systematic review and meta-ethnography from the perspective of the patients. PLoS ONE. 2012; 7(5):e37117 10.1371/journal.pone.0037117 22606338 PMC3351420

[5] Rosenfeld B. Methodological issues in assisted suicide and euthanasia research. Psychol Public Policy Law. 2000; 6:559–574. 12661541

[6] Chochinov HM, Wilson KG, Enns M, Lander S. Depression, Hopelessness, and suicidal ideation in the terminally ill. Psychosomatics. 1998; 39:366–370. 9691706 10.1016/S0033-3182(98)71325-8

[7] Kelly B, Burnett P, Pelusi D, Badger S, Varghese F, Robertson M. Terminally ill cancer patients' wish to hasten death. Palliat Med. 2002;16:339–345 12132547 10.1191/0269216302pm538oa

[8] Albert SM, Rabkin JG, Del Bene ML, Tider T, O'Sullivan I, Rowland LP, et al Wish to die in end-stage ALS. Neurology. 2005; 65:68–74. 16009887 10.1212/01.wnl.0000168161.54833.bb PMC1201540

[9] Rodin G, Zimmermann C, Rydall A, Jones J, Shepherd FA, Moore M, et al The desire for hastened death in patients with metastatic cancer. J Pain Symptom Manage. 2007; 33:661–675. 17531909 10.1016/j.jpainsymman.2006.09.034

[10] Mystakidou K, Rosenfeld B, Parpa E, Katsouda E, Tsilika E, Galanos A, et al Desire for death near the end of life: the role of depression, anxiety and pain. Gen Hosp Psychiatry. 2005; 27:258–262. 15993258 10.1016/j.genhosppsych.2005.02.004

[11] Villavicencio-Chavez C, Monforte-Royo C, Tomas-Sabado J, Porta-Sales J, Maier M, Balaguer A. Physical and psychological factors and the wish to hasten death in advanced cancer patients. Psychooncology. 2014; 23:1125–1132. 10.1002/pon.3536 24706522

[12] Ohnsorge K, Gudat H, Rehmann-Sutter C. What a wish to die can mean: reasons, meanings and functions of wishes to die, reported from 30 qualitative case studies of terminally ill cancer patients in palliative care. BMC Palliat Care. 2014; 13:38 10.1186/1472-684X-13-38 25161387 PMC4144684

[13] Ohnsorge K, Gudat Keller HR, Widdershoven GA, Rehmann-Sutter C. 'Ambivalence' at the end of life: how to understand patients' wishes ethically. Nurs Ethics. 2012; 19:629–641. 22990424 10.1177/0969733011436206

[14] Coyle N, Sculco L. Expressed desire for hastened death in seven patients living with advanced cancer: a phenomenologic inquiry. Oncol Nursing Forum. 2004; 31:699–709. 10.1188/04.ONF.699-709 15252426

[15] Nissim R, Gagliese L, Rodin G. The desire for hastened death in individuals with advanced cancer: a longitudinal qualitative study. Soc Sci Med. 2009; 69:165–171. 10.1016/j.socscimed.2009.04.021 19482401

[16] Schroepfer TA. Mind frames towards dying and factors motivating their adoption by terminally ill elders. J Gerontol. 2006; 61:S129–39. 10.1093/geronb/61.3.s129 16670190

[17] Brown JH, Henteleff P, Barakat S, Rowe CJ. Is it normal for terminally ill patients to desire death? Am J Psychiatry. 1986; 143:208–211 3946656 10.1176/ajp.143.2.208

[18] Chochinov HM, Wilson KG, Enns M, Mowchun N, Lander S, Levitt M, et al Desire for death in the terminally ill. Am J Psychiatry. 1995;152:1185–1191. 7625468 10.1176/ajp.152.8.1185

[19] Hudson PL, Kristjanson LJ, Ashby M, Kelly B, Schofield P, Hudson A, et al Desire for hastened death in patients with advanced disease and the evidence base of clinical guidelines: a systematic review. Palliat Med. 2006; 20:693–701. 17060268 10.1177/0269216306071799

[20] Rosenfeld B. Assisted Suicide and the Right to Die: The Interface of Social Science, Public Policy, and Medical Ethics. New York, USA, American Psychological Association, 2004.

[21] Lavery JV, Boyle J, Dickens BM, Maclean H, Singer PA. Origins of the desire for euthanasia and assisted suicide in people with HIV-1 or AIDS: a qualitative study. Lancet. 2001; 358:362–367. 11502315 10.1016/S0140-6736(01)05555-6

[22] Mak YY, Elwyn G. Voices of the terminally ill: uncovering the meaning of desire for euthanasia. Palliat Med. 2005; 19:343–350 15984507 10.1191/0269216305pm1019oa

[23] Pearlman RA, Hsu C, Starks H, Back AL, Gordon JR, Bharucha AJ, et al Motivations for physician-assisted suicide. J Gen Intern Med. 2005; 20:234–239. 15836526 10.1111/j.1525-1497.2005.40225.x PMC1490083

[24] Rurup ML, Pasman HR, Goedhart J, Deeg DJH, Kerkhof AJFM, Onwuteaka-Philipsen BD. Understanding why older people develop a wish to die: a qualitative interview study. Crisis. 2011; 32:204–216. 10.1027/0227-5910/a000078 21940258

[25] Malpas PJ, Mitchell K, Johnson MH. "I wouldn't want to become a nuisance under any circumstances"—a qualitative study of the reasons some healthy older individuals support medical practices that hasten death. N Z Med J. 2012; 125:9–19. 22864152

[26] Raijmakers NJ, van der Heide A, Kouwenhoven PS, van Thiel GJ, van Delden JJ, Rietjens JA. Assistance in dying for older people without a serious medical condition who have a wish to die: a national cross-sectional survey. J Med Ethics. 2015; 41:145–150. 10.1136/medethics-2012-101304 24335917

[27] Gastmans C, Van Neste F, Schotsmans P. Facing requests for euthanasia: a clinical practice guideline. J Med Ethics. 2004; 30:212–217. 15082821 10.1136/jme.2003.005082 PMC1733828

[28] Dierckx de Casterlé B, Verpoort C, De Bal N, Gastmans C. Nurses' views on their involvement in euthanasia: a qualitative study in Flanders (Belgium). J Med Ethics. 2006; 32:187–192. 16574869 10.1136/jme.2005.011783 PMC2565778

[29] Kissane DW, Clarke DM, Street AF. Demoralization syndrome—a relevant psychiatric diagnosis for palliative care. J Palliat Care. 2001; 17:12–21. 11324179

[30] Kelly B, Burnett P, Pelusi D, Badger S, Varghese F, Robertson M. Factors associated with the wish to hasten death: a study of patients with terminal illness. Psychol Med. 2003; 33:75–81. 12537038 10.1017/s0033291702006827

[31] McClain-Jacobson C, Rosenfeld B, Kosinski A, Pessin H, Cimino JE, Breitbart W. Belief in an afterlife, spiritual well-being and end-of-life despair in patients with advanced cancer. Gen Hosp Psychiatry. 2004; 26:484–486. 15567216 10.1016/j.genhosppsych.2004.08.002

[32] Morita T, Sakaguchi Y, Hirai K, Tsuneto S, Shima Y. Desire for death and request to hasten death of Japanese terminally ill cancer patients receiving specialized inpatient palliative care. J Pain Symptom Manage. 2004; 27:44–52. 14711468 10.1016/j.jpainsymman.2003.05.001

[33] Chochinov HM, Hack T, Hassard T, Kristjanson LJ, McClement S, Harlos M. Understanding the will to live in patients nearing death. Psychosomatics. 2005; 46:7–10. 15765815 10.1176/appi.psy.46.1.7

[34] Johansen S, Holen JC, Kaasa S, Loge JH, Materstvedt LJ. Attitudes towards, and wishes for, euthanasia in advanced cancer patients at a palliative medicine unit. Palliat Med. 2005; 19:454–460. 16218157 10.1191/0269216305pm1048oa

[35] Van de Ven AH, Delbecq AL. The nominal group as a research instrument for exploratory health studies. Am J Public Health. 1972; 62:337–342. 5011164 10.2105/ajph.62.3.337 PMC1530096

[36] Williams PL, Webb C. The Delphi technique: a methodological discussion. J Adv Nurs. 1994;19:180–186. 8138622 10.1111/j.1365-2648.1994.tb01066.x

[37] Powell C: The Delphi technique: myths and realities. J Adv Nurs. 2003; 41:376–382 12581103 10.1046/j.1365-2648.2003.02537.x

[38] Tastle WJ, Wierman MJ. Using Consensus to measure weighted targeted agreement. Proceedings of the North American Fuzzy Information processing society conference, San Diego, USA, 2007.

[39] Cassell EJ. The nature of suffering and the goals of medicine. The New England Journal of Medicine, 1982:639–45. 7057823 10.1056/NEJM198203183061104

[40] World Health Organization. WHO. Available: http://www.who.int/cancer/palliative/definition/en/

[41] van Wijngaarden E, Leget C, Goossensen A. Experiences and motivations underlying wishes to die in older people who are tired of living: a research area in its infancy. Omega (Westport). 2014; 69:191–216. 25223313 10.2190/OM.69.2.f

[42] Mishara BL. Synthesis of research and evidence on factors affecting the desire of terminally ill or seriously chronically ill persons to hasten death. Omega (Wesport). 1999; 39:1–70. 10.2190/5YED-YKMY-V60G-L5U5 11657878

[43] Owen C, Tennant C, Levi J, Jones M. Suicide and euthanasia: patient attitudes in the context of cancer. Psychooncology. 1992; 1:79–88.

[44] Harris EC, Barraclough B. Suicide as an outcome for mental disorders. A meta-analysis. Br J Psychiatry. 1997; 170:205–228. 9229027 10.1192/bjp.170.3.205

[45] Van Loon RA. Desire to die in terminally ill people: a framework for assessment and intervention. Health Soc Work. 1999; 24:260–268. 10605631 10.1093/hsw/24.4.260

[46] Bruce A, Boston P. Relieving existential suffering through palliative sedation: discussion of an uneasy practice. J Adv Nurs. 2011; 67:2732–2740. 10.1111/j.1365-2648.2011.05711.x 21627682

[47] Kissane DW. The relief of existential suffering. Arch Intern Med. 2012; 172:1501–1505. 10.1001/archinternmed.2012.3633 22945389

[48] Rodríguez-Mañas L, Féart C, Mann G, Viña J, Chatterji S, Chodzko-Zajko W, et al Searching for an operational definition of frailty: a Delphi method based consensus statement: the frailty operative definition-consensus conference project. J Gerontol A Biol Sci Med Sci. 2013; 68:62–67. 10.1093/gerona/gls119 22511289 PMC3598366

[49] Cresswell KM, Panesar SS, Salvilla SA, Carson-Stevens A, Larizgoitia I, Donaldson LJ, et al Global research priorities to better understand the burden of iatrogenic harm in primary care: an international Delphi exercise. PLoS Med. 2013; 10(11):e1001554 10.1371/journal.pmed.1001554 24260028 PMC3833831

[50] Simon ST, Weingartner V, Higginson IJ, Voltz R, Bausewein C. Definition, categorization, and terminology of episodic breathlessness: consensus by an international Delphi survey. J Pain Symptom Manage. 2014; 47:828–838. 10.1016/j.jpainsymman.2013.06.013 24095285

[51] Sanduleanu S, le Clercq CM, Dekker E, Meijer GA, Rabeneck L, Rutter MD, et al Definition and taxonomy of interval colorectal cancers: a proposal for standardising nomenclature. Gut. 2014; pii: gutjnl-2014-307992. 10.1136/gutjnl-2014-307992 25193802

[52] Gordon M, Baker P, Catchpole K, Darbyshire D, Schocken D. Devising a consensus definition and framework for non-technical skills in healthcare to support educational design: A modified Delphi study. Med Teach. 2014; 22:1–6. 10.3109/0142159X.2014.959910 25244065

[53] Viola R: Intractable wish to die: Multiple questions about a challenging issue. J Palliat Care. 2014; 30:294–297. 25962264

