# Correction: An International Consensus Definition of the Wish to Hasten Death and Its Related Factors

**DOI:** 10.1371/journal.pone.0196754

**Published:** 2018-04-26

**Authors:** Albert Balaguer, Cristina Monforte-Royo, Josep Porta-Sales, Alberto Alonso-Babarro, Rogelio Altisent, Amor Aradilla-Herrero, Mercedes Bellido-Pérez, William Breitbart, Carlos Centeno, Miguel Angel Cuervo, Luc Deliens, Gerrit Frerich, Chris Gastmans, Stephanie Lichtenfeld, Joaquín T. Limonero, Markus A. Maier, Lars Johan Materstvedt, María Nabal, Gary Rodin, Barry Rosenfeld, Tracy Schroepfer, Joaquín Tomás-Sábado, Jordi Trelis, Christian Villavicencio-Chávez, Raymond Voltz

There are errors in the Funding section. The correct funding information is as follows: This work was supported by the Instituto de Salud Carlos III (grant number: PI14/00263) and the European Regional Development Fund (FEDER): Main researcher Albert Balaguer; Other researchers: Cristina Monforte-Royo, Joaquín Tomás-Sábado, Christian Villavicencio-Chávez, Mercedes Bellido-Pérez; aecc-Catalunya contra el Càncer of Barcelona (Investigació 2014): Main researcher: Albert Balaguer; Other researchers: Cristina Monforte-Royo, Josep Porta-Sales, Joaquín Tomás-Sábado, Christian Villavicencio-Chávez, Mercedes Bellido-Pérez, Amor Aradilla-Herrero; WeCare Chair: End-oflife care at the Universitat Internacional de Catalunya and ALTIMA: Main researchers: Cristina Monforte-Royo; Josep Porta-Sales; Albert Balaguer. The funders had no role in study design, data collection and analysis, decision to publish, or preparation of the manuscript.
